# ASPM and microcephalin expression in epithelial ovarian cancer correlates with tumour grade and survival

**DOI:** 10.1038/bjc.2011.117

**Published:** 2011-04-19

**Authors:** A Brüning-Richardson, J Bond, R Alsiary, J Richardson, D A Cairns, L McCormack, R Hutson, P Burns, N Wilkinson, G D Hall, E E Morrison, S M Bell

**Affiliations:** 1 Section of Ophthalmology and Neurosciences, Leeds Institute of Molecular Medicine, Welcome Trust Brenner Building, St James's University Hospital, Leeds LS9 7TF, UK; 2 Section of Oncology and Clinical Research, Leeds Institute of Molecular Medicine, St James's University Hospital, Leeds LS9 7TF, UK; 3 St James's Institute of Oncology, St James's University Hospital, Leeds LS9 7TF, UK

**Keywords:** epithelial ovarian cancer, ovarian ascites, ASPM, microcephalin, biomarkers

## Abstract

**Background:**

The clinico-pathological and molecular heterogeneity of epithelial ovarian cancer (EOC) complicates its early diagnosis and successful treatment. Highly aneuploid tumours and the presence of ascitic fluids are hallmarks of EOC. Two microcephaly-associated proteins, abnormal spindle-like microcephaly-associated protein (ASPM) and microcephalin, are involved in mitosis and DNA damage repair. Their expression is deregulated at the RNA level in EOC. Here, ASPM and microcephalin protein expression in primary cultures established from the ascites of patients with EOC was determined and correlated with clinical data to assess their suitability as biomarkers.

**Methods:**

Five established ovarian cancer cell lines, cells derived from two benign ovarian ascites samples and 40 primary cultures of EOC derived from ovarian ascites samples were analysed by protein slot blotting and/or immunofluorescence to determine ASPM and microcephalin protein levels and their cellular localisation. Results were correlated with clinico-pathological data.

**Results:**

A statistically significant correlation was identified for ASPM localisation and tumour grade, with high levels of cytoplasmic ASPM correlating with grade 1 tumours. Conversely, cytoplasmic microcephalin was only identified in high-grade tumours. Furthermore, low levels of nuclear microcephalin correlated with reduced patient survival.

**Conclusion:**

Our results suggest that ASPM and microcephalin have the potential to be biomarkers in ovarian cancer.

Epithelial ovarian cancer (EOC) remains one of the deadliest gynaecological cancers with more than 204 000 new cases being diagnosed worldwide each year (Cancer Research UK, 2010). The cancer is characterised by presentation with advanced disease, a high rate of recurrence despite response to primary surgery/chemotherapy and ultimately low survival; a median overall survival of 3–4 years was defined in a recent international phase III clinical trial (Bookman *et al*, 2009). There is a need to better characterise these tumours to identify markers of poor prognosis, which may be used to guide subsequent management.

A consistent clinical feature of advanced EOC is the accumulation of ascitic fluid in the peritoneum of patients. Large volumes of fluid from patients with newly diagnosed or recurrent EOC can be taken at the time of primary surgery or therapeutic paracentesis and have been shown to be a useful source of malignant ovarian cancer cells for the study of EOC with regards to immunology, gene expression and drug development (Parella *et al*, 2003; Graves *et al*, 2004; Bamias *et al*, 2007). The genes *MCPH5* encoding abnormal spindle-like microcephaly-associated protein (ASPM) and *MCPH1* encoding microcephalin have been shown to be associated with autosomal primary microcephaly, a developmental brain disorder characterised by an architecturally normal but small brain and associated mental retardation (Aicardi, 1998; Bond *et al*, 2002, 2003; Jackson *et al*, 2002). In interphase, ASPM has a predominantly centrosomal or nuclear localisation (Zhong *et al*, 2005; Higgins *et al*, 2010). It has an important role in mitosis while localised to the pericentrosomal matrix surrounding the mitotic spindle (Fish *et al*, 2006; Higgins *et al*, 2010). Microcephalin is involved in DNA damage repair and a cell cycle control pathway that prevents premature entry into mitosis (Trimborn *et al*, 2004; Rai *et al*, 2006). It has a reported centrosomal localisation (Zhong *et al*, 2006; Tibelius *et al*, 2009), while a nuclear localisation has been reported in immunofluorescence (Rai *et al*, 2006) and immunohistochemistry (IHC; Rai *et al*, 2006, Human Protein Atlas (http://www.proteinatlas.org)). We hypothesised that as mitosis- and DNA repair-associated proteins, ASPM and microcephalin, might have a role in the tumourigenesis of cancers with highly aneuploid tumours, such as high-grade EOC. Recently, both ASPM and microcephalin have been shown to be deregulated in a variety of cancers (Bartek, 2006; Horvath *et al*, 2006; Hagemann *et al*, 2008; Kabbarah *et al*, 2010; Richardson *et al*, 2010). Publications relating specifically to EOC have demonstrated an upregulation of mRNA levels for ASPM and a decrease for microcephalin (Kouprina *et al*, 2005; Rai *et al*, 2006). We investigated ASPM and microcephalin in established ovarian cancer cell lines and in primary cultures derived from ovarian ascites to determine whether their expression is altered at the protein level in this cancer. We then ascertained whether correlations with associated clinical data could be useful prognostic markers in EOC. Here, we demonstrate for the first time that ASPM and microcephalin protein expression is deregulated in primary cultures of malignant cells obtained from ascitic fluids of EOC patients and that this expression shows an association with survival or tumour grade. Potentially, both ASPM and microcephalin may be useful biomarkers for EOC.

## Materials and methods

### Patient samples and cell lines

The sample panel consisted of five established ovarian cancer cell lines (1847, TRI75, SKOV-3, JAMA-2 and OVCA433), all obtained from Cancer Research UK, and cells from 40 primary cultures of EOC derived from ovarian ascites samples collected from patients receiving treatment at St James's Hospital Institute of Oncology. Also included were primary cultures established from the peritoneal washings of two patients who were found to have benign epithelial neoplasms (1153-A1, serous cystadenoma and 1078-A1, mucinous cystadenoma). Ethical approval was obtained for all clinical samples involved in this study from the local Research Ethics committee of the Leeds Teaching Hospitals NHS Trust, Leeds.

### Establishment of primary cultures from ovarian ascites

On receipt, ascites samples were processed aseptically as described previously (Ingram *et al*, 2010). Briefly, ascites were centrifuged at 2000 r.p.m. to separate the cells from the ascitic supernatant. The decanted supernatant was reserved for use as a cell culture supplement. Contaminating red blood cells were removed by 10 min treatment with red blood cell lysing buffer (Sigma-Aldrich, Poole, UK) according to manufacturers’ instructions. Cell pellets were resuspended in RPMI 1640 medium supplemented with 10% FCS and, additionally at passage (p) 0, with 10% decanted autologous supernatant, and were incubated in 25–75 cm^2^ flasks in a 5% CO_2_ incubator at 37°C. Once cells were confluent they were trypsinised and passaged at a 1/3 dilution. Passage levels were kept low to minimise phenotypic changes due to adaptation to cell culture.

### Antibodies

The rabbit affinity-purified ASPM antibody 216-1 and the rabbit affinity-purified microcephalin antibody (Abcam, Cambridge, UK ab2162) were used throughout this study. Although information on the specificity of these antibodies has been previously published (Xu *et al*, 2004; Higgins *et al*, 2010), we further confirmed the specificity of the immunofluorescence staining by pre-incubation with peptides for both antibodies (Supplementary Figures 1A–C) and by siRNA for microcephalin (Supplementary Figure 1D).

### Slot blotting

To compare ASPM protein levels in established cancer cell lines, benign samples and primary cultures, a slot blotting technique was applied. Both primary cultures and cell lines were grown in 75 cm^2^ flasks until confluent. The cells were washed with PBS. Fresh PBS (10 ml) was added and the cells were detached using cell scrapers. The cells were pelleted by centrifugation for 5 min at 1000 r.p.m., resuspended in 1 ml of ice-cold RIPA buffer (50 mM Tris-HCL pH 8, 150 mM NaCl, 1% NP-40, 0.5% sodium deoxycholate, 0.1% SDS, 5 mM EDTA and Roche ‘Complete’ protease inhibitor cocktail (Roche, Welwyn Garden City, UK)) and transferred to Eppendorf tubes. To promote lysis the cells were passed repeatedly through a 19-gauge needle after which the samples were placed on ice for 15 min before being transferred to a microcentrifuge and spun for 30 min at 13 000 r.p.m. at 4°C. The supernatants were carefully removed and aliquots were stored at −20°C until further use. Protein estimation was performed using the Modified Bradford method (Bio-Rad protein assay, Bio-Rad Laboratories, Hemel Hempstead, UK). Samples with a total protein concentration of 0.5 mg ml^−1^ or more were analysed by slot blotting including 4 cell lines, 1 benign sample and 32 primary cultures.

For slot blotting, protein lysates were diluted in RIPA buffer to a concentration of 0.1 mg ml^−1^ of protein and heated to 95°C for 5 min. A volume of 100 *μ*l of the diluted cell lysate solution was loaded onto nitrocellulose supported by a pre-wetted Whatman filter paper (Whatman International Ltd, Maidstone, UK) contained within a slot blot apparatus (SCIE-PLAS, Cambridge, UK) according to the manufacturers’ instructions. Lysate protein was transferred onto the nitrocellulose in individual slots by applying a vacuum of 0.8 atmospheres for 2 min. All slots were washed with 100 *μ*l of PBS with pressure reapplied for 2 min. The nitrocellulose was removed from the slot blotter apparatus and blocked with 1% non-fat dried milk in PBS for 30 min on a rocker at room temperature. Rabbit anti-ASPM antibody 217-2 (Higgins *et al*, 2010) at 1/200 in non-fat dried milk in PBS or rat anti-tyrosinated *α*-tubulin (endogenous control, Transduction Laboratories, Lexington, KY, USA) at 1/3000 was added to the nitrocellulose and incubated for 1 h at room temperature. After three washes for 5 min each in PBS-Tween (0.1%) the nitrocellulose was incubated in the secondary antibody solutions, anti-rabbit or anti-rat IgG-horseradish peroxidase (Pierce ECL Femto kit, Pierce, Cramlington, Northumberland, UK) at 1/50 000 in non-fat dried milk in PBS for another hour on a rocker at room temperature. The nitrocellulose was washed three times for 5 min in PBS-Tween and once in PBS only. The Pierce SuperSignal West Femto ECL reagent (Pierce) was added to the nitrocellulose membrane and incubated for 5 min. Signals were recorded using a Bio-Rad ChemiDoc XRS system (Bio-Rad). The slot blot images from three repeats for each sample were scanned and band densities analysed to determine relative levels of ASPM and of tyrosinated *α*-tubulin using Bio-Rad Quantity 1 software (Bio-Rad). After background levels were subtracted the resulting values were examined as a ratio of ASPM/*α*-tubulin for each sample, with high values representing the samples with highest ASPM protein levels. Cutoff points were determined to classify samples as having low, medium or high ASPM levels.

### Indirect immunofluorescence

Primary cultures and cell lines were grown on sterile coverslips in 6-well tissue culture dishes (Nunc, Thermo Fisher Scientific, Roskide, Denmark) until they reached at least 75% confluency. The cells were washed in PBS and fixed with ice cold 100% methanol at −20°C for 2 min. After removal of the methanol, the cells were incubated in 0.5% non-fat dried milk in PBS for 10 min to block non-specific antibody-binding sites. The rabbit anti-ASPM antibody 216-1 (1/500) (Higgins *et al*, 2010), the rabbit anti-microcephalin antibody (1/100, Abcam, ab2612) and a rat anti-*α*-tubulin antibody (1/500, Serotec, Kidlington, UK) were prepared in blocking solution (0.5% non-fat dried milk in PBS). They were spun for 5 min in a microfuge at 13 000 r.p.m. before use. Coverslips were inverted over a 200 *μ*l drop of this supernatant on parafilm in a humidified chamber for 1 h at room temperature, then returned to the 6-well dish for washing. After three washes in PBS for 5 min each, the coverslips were incubated in secondary antibody solution containing a mixture of goat anti-rabbit IgG Alexa fluor 488 (1/500, Molecular Probes, Invitrogen, Paisley, UK), goat anti-rat IgG Alexa fluor 568 (1/500, Molecular Probes) and DAPI (5 *μ*g ml^−1^) diluted with the blocking solution and spun for 5 min at 13 000 r.p.m. before introduction to the cells. After a further incubation for 1 h in a humidified chamber at room temperature, the coverslips were washed four times in PBS for 5 min each and then mounted onto microscope slides using Mowiol (Sigma). Slides were allowed to set overnight and viewed using a Zeiss Axiovert 200 fluorescence microscope (Zeiss, Welwyn Garden City, UK) with a × 63 Plan Apo oil immersion lens. Images were captured and processed using a Hamamatsu Orca ER cooled CCD camera (Hamamatsu, Hamamatsu City, Japan) and Andor IQ imaging software (Belfast, UK).

### Determination of protein levels in primary cultures and cell lines by immunofluorescence

We determined the levels and localisation of ASPM (*n*=22) and microcephalin immunofluorescence (*n*=36) in the cell lines and in the primary cultures for comparison with the associated clinical data. Five hundred to one thousand cells were observed for staining profiles and quantification of fluorescence intensities was performed for each sample in each of 20 interphase cells and at least 20 mitotic cells using Andor IQ software. Samples containing <20 mitotic cells per 1000 cells were not included in the mitotic analysis. For microcephalin, nuclei were identified by DAPI staining and the fluorescence within the nucleus measured and divided by the area to give the integrated intensity per unit area. The same method was applied for establishing nuclear ASPM and cytoplasmic fluorescence levels. For ASPM levels in mitotic cells, the fluorescence intensity within a defined region covering each spindle pole at metaphase was recorded and the sum of intensities for both spindle poles was determined. All recorded data were examined and subsequently presented as weak, medium or strong after establishing logical cutoff points for each category.

### Statistical analysis of slot blot and fluorescence data

Associations between clinical variables and ASPM or microcephalin proteins in the primary cultures were undertaken using Fisher's Exact test for categorical variables (site, disease stage, grade and morphology at presentation) and the non-parametric Kruskall–Wallis and Wilcoxon–Mann–Whitney test for continuous variables. The association between ASPM or microcephalin and survival data was evaluated using the Kaplan–Meier method of estimating survival functions and Cox proportional hazards regression. All analysis was undertaken in the R Environment for Statistical Computing (R Design Core Team, Vienna, Austria).

## Results

### Patient data

Clinical data was available for 40 ovarian cancer patients from which ascitic fluid was obtained to generate the primary cultures. The mean age of the patient group was 62.2 years with a range of 26–85 years. In all, 35 out of 40 (87.5%) of the patients were over 50 years of age and 5 out of 40 (12.5%) were under 50 years of age. Among the ovarian cancer patients, 3 out of 40 (7.5%) presented with stage 1 ovarian cancer, 1 out of 40 (2.5%) stage 2, 30 out of 40 (75%) stage 3 and 6 out of 40 (15%) stage 4 disease. Grading and tumour typing was performed on histological material according to the FIGO grading system identifying 8 out of 40 (20%) of patients with grade 1 tumours, 11 out of 40 (27.5%) with grade 2 tumours and 19 out of 40 (47.5%) with grade 3 tumours. One sample was a borderline case (2.5%). One patient (2.5%) had been given chemotherapy based on ascitic fluid cytology together with elevated tumour markers (CA125, 1607; CEA<1.0) and radiological features consistent with stage 3c primary peritoneal carcinoma. It was not possible to grade this tumour based on the cytological preparation. The majority of the carcinomas were high-grade serous adenocarcinoma (33 out of 40, 82.5%), with the remaining samples comprising low-grade serous cystadenocarcinoma (2 out of 40, 5%), endometrioid and mucinous type (1 out of 40 each, 2.5%) and a further group assigned serous carcinoma (2 out of 40, 5%), which were post-chemotherapy samples. The median survival times from diagnosis for the patients was 39 months with a range of 2.7–112.5 months.

### ASPM expression determined by slot blotting

**Figure 1:**
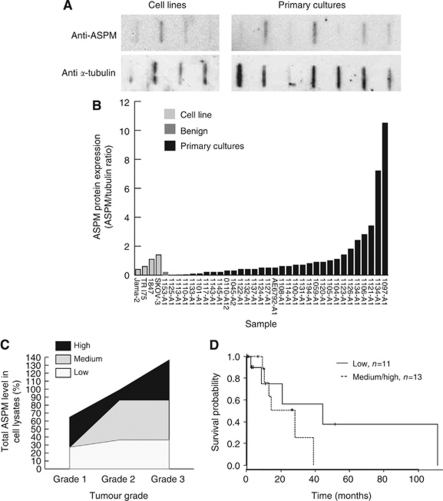
Abnormal spindle-like microcephaly-associated protein protein levels differ among primary cultures. (**A**) Slot blot profile of four established ovarian cell lines (top left) and seven primary cultures of EOC (top right). Each band represents one sample (10 *μ*g) probed with the ASPM antibody 217-2. Bottom row shows bands obtained for *α*-tubulin in the samples. (**B**) Slot blot profile for ASPM after normalisation against *α*-tubulin revealing a range of ASPM protein levels among established cell lines, one benign sample (1153-A1) and primary cultures (*n*=37). (**C**) ASPM levels increase with tumour grade. (**D**) Kaplan–Meier survival plot showing longer survival for groups with low ASPM levels in comparison with medium/high ASPM levels.

Previous publications have indicated that ASPM levels are upregulated in various cancers at the RNA level (Kouprina *et al*, 2005; Lin *et al*, 2008). Our own Affymetrix gene expression array data from a previous study composed of primary cultures derived from ovarian ascites samples and their associated solid tumours showed that mRNA expression levels differed significantly among the samples (Supplementary Figure 2). A correlation between expression in matched tumour and ascites samples was also shown (ASPM correlation coefficient 0.6, microcephalin correlation coefficient 0.43; Supplementary Figure 3). We wanted to assess if such differences were reflected in protein levels using our ascites data set and ovarian cancer cell lines. Analysis of the slot blot data for ASPM revealed that there were differences in the ASPM protein levels among the different ovarian cancer cell lines (Figures 1A and B). JAMA-2 had the lowest expression of the four established cell lines followed by TRI75 and 1847, with SKOV-3 having the highest (Figure 1B). Variation was also observed among the primary cultures. The lysate from a benign cystadenoma case (control, sample 1153-A1) had one of the lowest levels of ASPM protein in comparison to the other primary cell culture lysates (Figure 1B). Analysis of the data revealed that with increasing tumour grade there was an increase in total ASPM protein in the sample set (Figure 1C). Association studies of ASPM levels and mitotic activity within the primary cultures showed no statistically significant association of total ASPM levels with the number of cells in mitosis. This confirmed that the increase in ASPM levels with tumour grade was not simply due to an increase in mitotic activity in high-grade samples (Supplementary Figure 4A). To further validate these results, proliferation in tumour tissues from 18 patients with associated ascites was determined by Ki-67 IHC staining. Abnormal spindle-like microcephaly-associated protein levels as determined by slot blot of primary cell culture lysates were not associated with Ki-67-staining patterns in the primary tumour that generated the ascites (Supplementary Figures 4B and C). Patients with low ASPM levels had a longer median survival (44.6 months) than patients with high ASPM levels (28.2 months; Figure 1D). However, this observation was not significant at the nominal significant level (HR=2.030, 95% CI=(0.486, 8.473), *P*=0.331). In addition, our slot blot analysis demonstrated that there were significantly fewer deaths among patients with high ASPM levels (1 out of 11 9%) when compared with patients with low levels (7 out of 13 53.8% *P*=0.036).

### ASPM expression and localisation visualised by immunofluorescence in primary cultures

**Figure 2:**
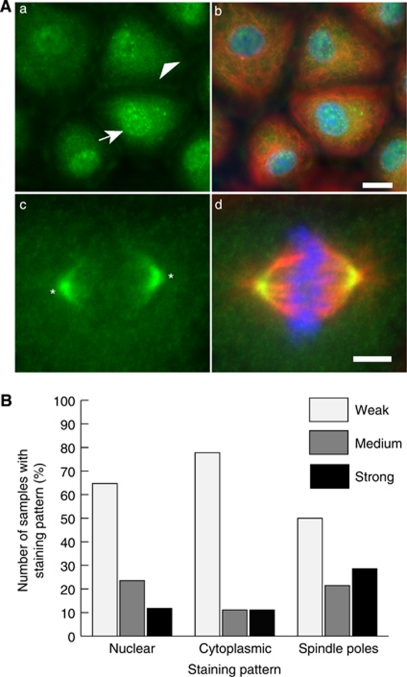
Immunofluorescence staining patterns of ASPM in primary cultures. (**A**) In interphase ASPM can be found in the cytoplasm and the nucleus (a, b; arrowhead indicates cytoplasmic and arrow indicates nuclear staining), whereas in mitosis ASPM localises at the spindle poles (c; asterix indicates spindle pole). Green staining indicates ASPM, red *α*-tubulin and blue DAPI. Scale bars are 10 *μ*m (b) and 5 *μ*m (d). (**B**) Histogram summarising staining patterns and frequencies observed for ASPM after fluorescence quantification.

**Figure 3:**
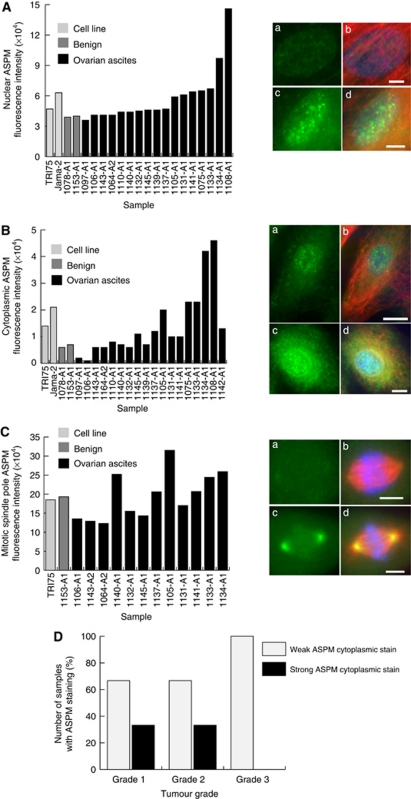
Established ovarian cell lines and primary cultures display different ASPM levels after fluorescence quantification. (**A**) Histogram illustrating the range of ASPM levels in the nucleus established by immunofluorescence (*n*=21). Examples of nuclei with low (a, b) and high (c, d) ASPM levels are shown. Green staining indicates ASPM, red *α* -tubulin and blue DAPI. Scale bars are 5 *μ*m. (**B**) Histogram illustrating the range of ASPM levels in the cytoplasm as established by immunofluorescence. Examples of low (a, b) and high (c, d) cytoplasmic ASPM levels are shown (*n*=22). Scale bars are 10 *μ*m (b) and 5 *μ*m (d). (**C**) Histogram illustrating the range of ASPM levels at the spindle poles as established by immunofluorescence (*n*=14). Examples of spindle poles with low (a, b) and high (c, d) ASPM levels are shown. Scale bar is 5 *μ*m. Green staining indicates ASPM, red *α*-tubulin and blue DAPI. (**D**) Graph depicting an association of cytoplasmic ASPM levels in primary cultures with tumour grade. High cytoplasmic ASPM levels are lost in high-grade tumours (*P*=0.0351).

Having established that at both the mRNA and protein levels there were differences in ASPM expression among established ovarian cancer cell lines and the primary cultures, we next investigated the cellular localisation and expression levels of ASPM using immunofluorescence. Staining with the ASPM antibody revealed that there was cytoplasmic and nuclear staining in interphase cells in all of the samples as well as spindle pole staining in mitotic cells (Figure 2A). However, the staining patterns and intensity differed among samples and were scored as weak/medium/strong expression at each localisation after quantifying fluorescence intensities. Staining patterns in two samples (1078-A1 and 1153-A1, primary cultures from peritoneal washes from patients with benign gynaecological conditions) revealed weak nuclear and cytoplasmic staining with medium ASPM labelling at the spindle poles (Supplementary Figure 5A). It seems likely that these observations represent typical ASPM localisations and expression levels in ‘normal’ control cells. The majority of the 18 tumour-derived samples had both weak nuclear and weak cytoplasmic staining (64.7 and 77.8%, respectively), whereas 50% of the samples had weak mitotic ASPM staining. Medium staining was observed for 23.5% (nuclear), 11.1% (cytoplasmic) and 21.4% (spindle poles) of cultures. Strong nuclear staining was observed in 11.8% of the cultures, strong cytoplasmic staining in 11.1% and strong spindle pole staining in 28.6% (Figure 2B). Analysis of the staining data indicated that the majority of the primary cultures had nuclear- and spindle pole-staining intensities similar to the intensities obtained for the established cell lines. Only two samples (1134-A1 and 1108-A1) for both nuclear staining and cytoplasmic staining and two samples (1140-A1 and 1105-A1) for spindle pole staining had markedly higher levels of ASPM than were observed in the cell lines (Figures 3A–C). Generally, levels of cytoplasmic staining were most variable among the primary cultures (Figure 3B). Statistical analysis for the 18 primary culture samples with associated clinical data indicated that strong cytoplasmic ASPM staining levels were significantly associated with a low tumour grade (*P*=0.0351; Figure 3D).

### Expression profiles and localisation of microcephalin in primary cultures

**Figure 4:**
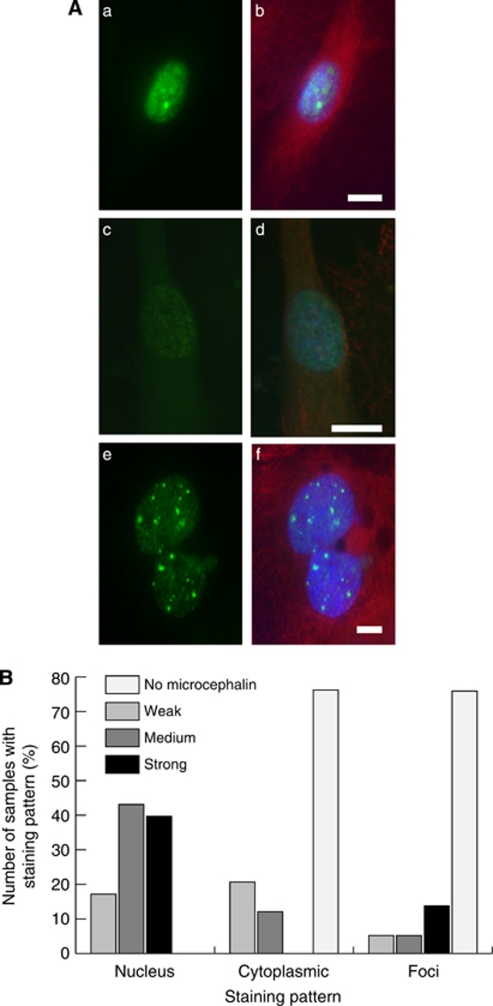
Immunofluorescence staining patterns of microcephalin in primary cultures. (**A**) In interphase microcephalin can be found in the nucleus (a, b) and the cytoplasm (c, d) and in nuclear foci (e, f). Scale bars are 10 *μ*m (b), 10 *μ*m (d) and 5 *μ*m (f). Green staining indicates microcephalin, red *α*-tubulin and blue DAPI. (**B**) Graph summarising staining patterns and frequencies observed for microcephalin staining after fluorescence quantification.

**Figure 5:**
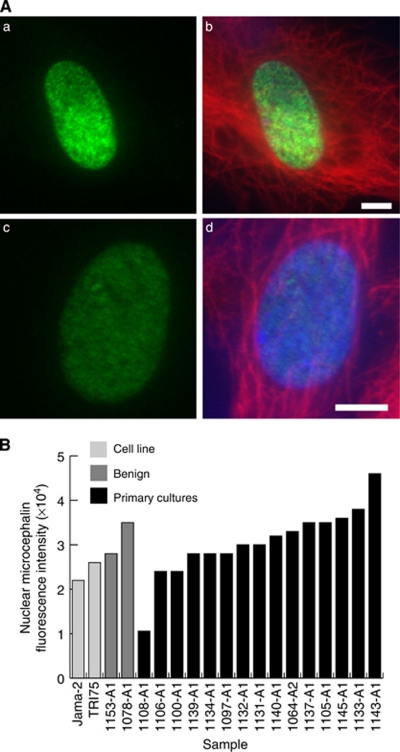
Established ovarian cell lines and primary cultures display a variety of microcephalin expression levels. (**A**) Examples of nuclei with high (a, c) and low (b, d) microcephalin levels are shown. Green staining indicates microcephalin, red *α*-tubulin and blue DAPI. Scale bars are 5 *μ*m. (**B**) Histogram illustrating the range of microcephalin expression in the nucleus established by immunofluorescence (*n*=19).

**Figure 6:**
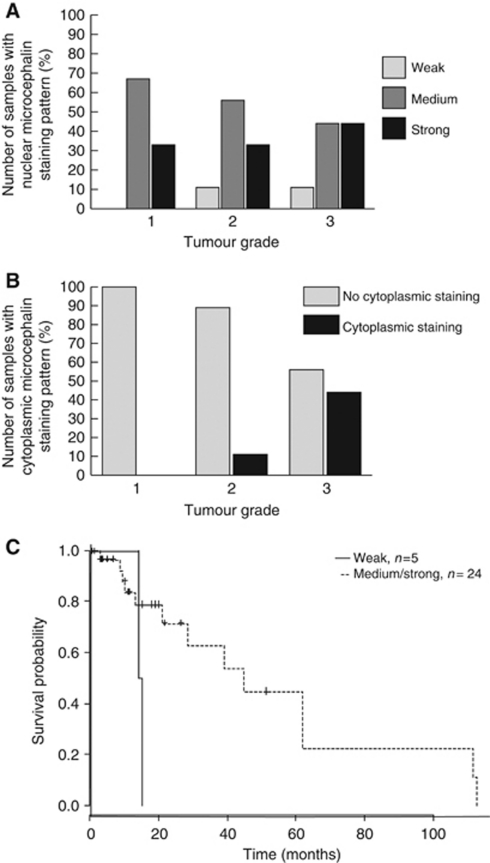
Nuclear and/or cytoplasmic microcephalin levels are associated with tumour grade and survival. (**A**) Weak microcephalin levels in the nucleus are associated with higher-grade tumours. (**B**) Cytoplasmic microcephalin is only detected in higher-grade tumours (*P*=0.047). (**C**) Kaplan–Meier survival plot showing survival for groups with weak nuclear microcephalin levels and medium/strong nuclear microcephalin levels (*P*=0.081).

Immunofluorescence staining with the microcephalin antibody on 36 samples revealed three distinct staining patterns: nuclear, cytoplasmic and nuclear foci. After establishing cutoff points following calculation of staining intensities, microcephalin levels in the nucleus were classified as weak, medium or strong for each sample. Overall, nuclear microcephalin expression in the majority of the samples fell into the medium or strong category. Cytoplasmic staining was seen in only 25% of the samples with all exhibiting a weak staining pattern. Twenty-five percent of the samples displayed strong multiple nuclear foci staining with the remaining samples displaying only one or two strong foci per nucleus. Figure 4 shows examples of each staining pattern (Figure 4A) and a summary of the microcephalin localisation pattern (Figure 4B). The two control samples, 1078-A1 and 1153-A1, displayed strong nuclear staining and strong foci staining was also detected in 1153-A1 (Supplementary Figure 5B). These are therefore likely to represent the ‘normal’ localisation and expression level of microcephalin in cultured ovarian epithelial cells. The most common staining pattern observed in the tumour-derived primary cultures was nuclear staining (Figure 5A); therefore, the fluorescence data from these observations were plotted for each individual primary culture sample in comparison to the established cell lines (Figure 5B). The majority of the samples had nuclear staining intensities similar to the cell lines with the exception of 1108-A1 (markedly lower) and 1143-A1 (higher). Clinical data were available for 32 primary cultures. Interestingly, weak nuclear staining was found only in the higher-grade tumours (moderately differentiated, grade 2, to poorly differentiated, high grade 3). In primary cultures of all patients with well-differentiated tumours (grade 1), medium or strong nuclear staining was observed (Figure 6A). An absence of cytoplasmic microcephalin immunostaining was observed in low/well differentiated (grade 1) tumours, whereas cytoplasmic staining correlated with grade 2 and 3 tumours exclusively (*P*=0.047; Figure 6B). This finding suggests that there is a positive correlation between the aberrant cytoplasmic localisation of microcephalin and tumour development. No association was found between nuclear foci staining and the clinical data.

Statistical analysis of the immunofluorescence data revealed weak evidence of an association between survival and weak nuclear microcephalin staining pattern (HR=0.2311, 95% CI=(0.045, 1.201), *P*=0.081). The association indicates that patients with a weak score have an increased hazard of death, which correlates with the loss of nuclear staining and increase in cytoplasmic staining seen in the higher tumour grades (Figure 6C).

We also examined ASPM and microcephalin expression using IHC in 23 paraffin-embedded primary tumour samples from patients whose ascites were used to generate primary cultures. Importantly, staining patterns and intensities in these samples were similar to those detected in the corresponding primary cultures (Supplementary Figures 6A and B), confirming that the cultures were a viable proxy for tumour tissue samples in this study. In six cases, adjacent normal tissue was present, as well as tumour, providing useful controls.

## Discussion

Epithelial ovarian cancer is a very heterogeneous disease. Histologically there are at least four subtypes. The most common subtype, serous carcinoma, can be classified on a molecular basis into two major groups (type I, low-grade carcinomas and type II, high-grade carcinomas) according to p53 (high-grade carcinomas; Ahmed *et al*, 2010) or KRAS/BRAF (low-grade carcinomas) mutations (Cho and Shih, 2009). Prognosis for patients with stage 1 disease ovarian cancers is generally very good and 5-year survival rates are more than 90%. However, most ovarian cancers are detected at an advanced stage resulting in a 5-year survival rate of <25%. Chemoresistance is a common occurrence after chemotherapy and tumour debulking. Furthermore, disease incidence in women over 65 has increased by more than 40% since the 1970s (Cancer Research UK, 2010). Therefore, there is a great demand for biomarkers and new therapeutics to manage EOC. The aim of our study was multifold. First, we wanted to establish if the two *MCPH* gene products, ASPM and microcephalin, were deregulated in EOC at the protein level. We also aimed to investigate if there was an association between tumourigenesis and ASPM and microcephalin protein levels in EOC, because ovarian cancers exhibit a high degree of aneuploidy and these proteins are known to be involved in mitotic processes and DNA repair. There has recently been an increased interest in proteins associated with chromosomal instability and aneuploidy in cancer. High expression of the mitotic kinase BubR1 was demonstrated to be associated with aggressive clinico-pathological parameters including advanced stage of disease, serous histology and high-grade tumours in EOC (Lee *et al*, 2009). This protein was shown to be a prognostic marker for recurrence-free survival rates in EOC. Our final aim was therefore to examine whether ASPM and microcephalin might be similarly useful as biomarkers in this disease.

In our study, we utilised primary cultures of EOC derived from ascites. Malignant cells derived from ascitic fluids have recently been used for the study of drug response and novel treatment regimes (Ingram *et al*, 2010; Scarberry *et al*, 2010). Affymetrix gene expression array data showed that the mRNA levels of both ASPM and microcephalin varied between primary cultures grown from malignant ascites and correlated with levels observed in primary cultures derived from their associated primary tumours (Supplementary Figure 1). In the present study, these primary cultures were an ideal resource as we were able to clearly define intracellular localisations and quantify protein levels of ASPM and microcephalin in malignant cells, which is difficult to achieve by other techniques such as FACS or IHC on solid tumour samples. Analysis of ASPM in our primary cultures by slot blotting revealed that high ASPM levels were mainly seen in grade 3 tumour patients and were associated with reduced survival. Abnormal spindle-like microcephaly-associated protein and microcephalin immunofluorescence indicated that protein levels and localisations varied among the samples, though this data should be interpreted cautiously given the possibility that differently localised pools of protein might be differentially extracted by the immunostaining procedure. Nevertheless, variations in protein localisation appeared to be significant for tumour development. High levels of ASPM in the cytoplasm were associated with reduced tumour grade, whereas high nuclear levels of microcephalin and an absence of cytoplasmic microcephalin was associated with better survival and lower tumour grade. A previous publication showed that *MCPH1* mutations caused the localisation of microcephalin to change from nuclear to cytoplasmic, providing a plausible explanation for our latter observations (Wu *et al*, 2009). Interestingly, mutations of *BRCA1* in EOC have been shown to be responsible for a shift from nuclear to cytoplasmic localisation, inhibiting nuclear DNA repair and transcription function (Rodriguez *et al*, 2004). Similar effects may be seen with microcephalin mutation. Unfortunately, we were unable to consistently confirm a centrosomal localisation for microcephalin in our cultures using the reagents available to us (Supplementary Table 1), but we note that our conclusions are not incompatible with the biological functions of microcephalin at this organelle described by previous investigators (i.e., Tibelius *et al*, 2009).

In future studies it will be interesting to investigate associations between microcephalin and ASPM levels and chemoresistance to paclitaxel/carboplatin. Carboplatin reacts with DNA inhibiting DNA replication and causing DNA breaks, whereas paclitaxel is a mitosis-specific drug, binding specifically to microtubules and inhibiting mitosis. Both of these modes of action could potentially be impacted by changes in the functional activity of microcephalin or ASPM. In conclusion, we have shown that ASPM and microcephalin have variable expression levels in primary cultures of malignant cells derived from ovarian tumour ascites and that there are distinctive localisation patterns for both proteins. Our data indicate that ASPM and microcephalin may be considered as potential prognostic markers in ovarian cancer and further large-scale studies are warranted.

## Supplementary Material

Supplementary Figure 1

Supplementary Figure 1D
