# Supplementary figures and images for: ASPM and microcephalin expression in epithelial ovarian cancer correlates with tumour grade and survival
Source: Br J Cancer. 2011 Apr 19;104(10):1602–10. doi: 10.1038/bjc.2011.117 (PMC3101901; doi:10.1038/bjc.2011.117)

A

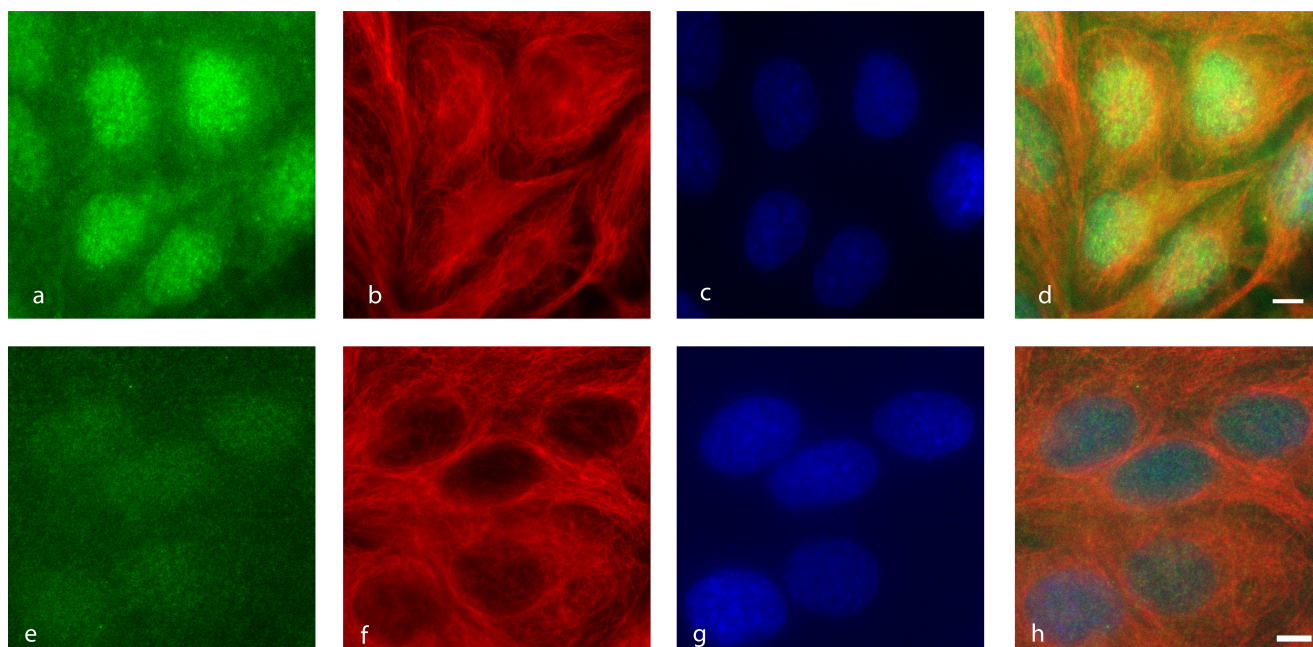

B

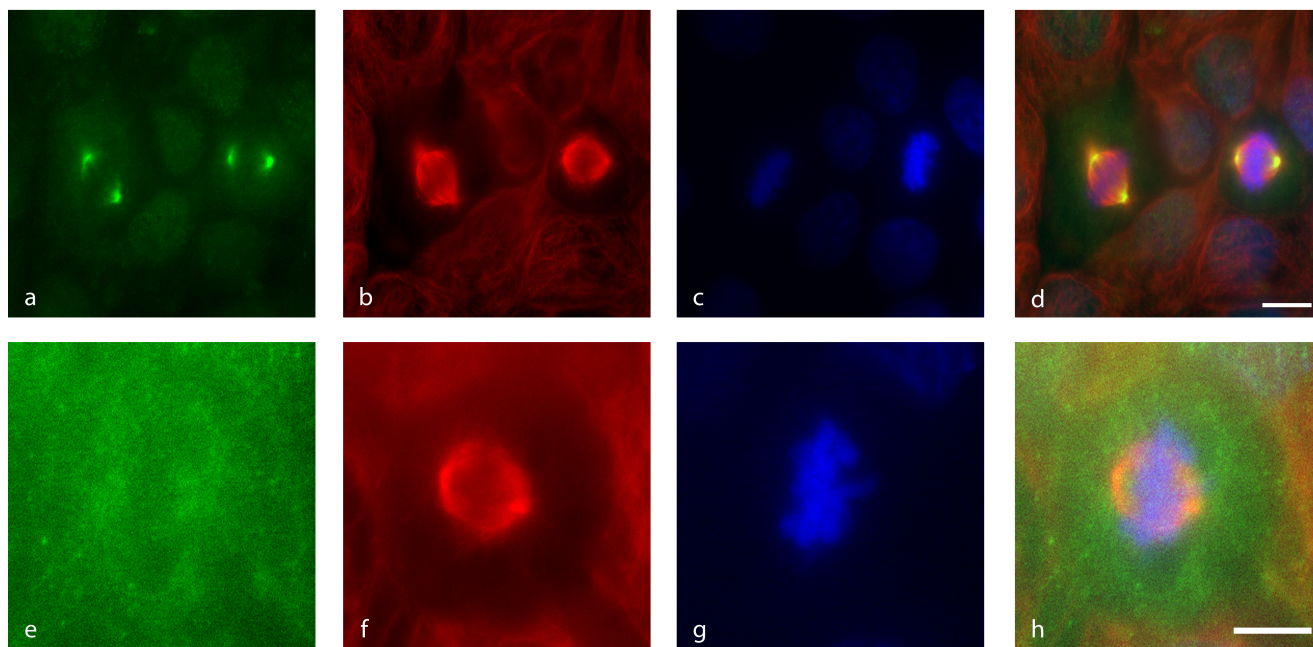

C

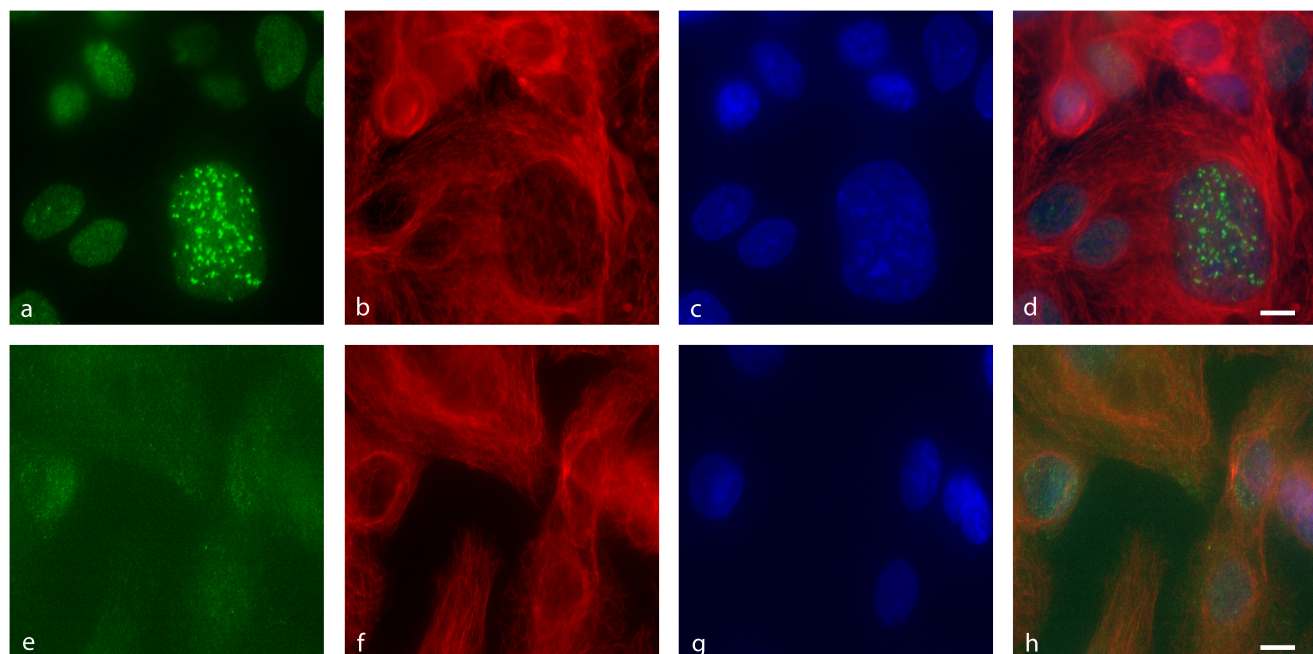

Supplementary Figure 1

Supplement: Supplementary Figure 1 [file bjc2011117x1.pdf]

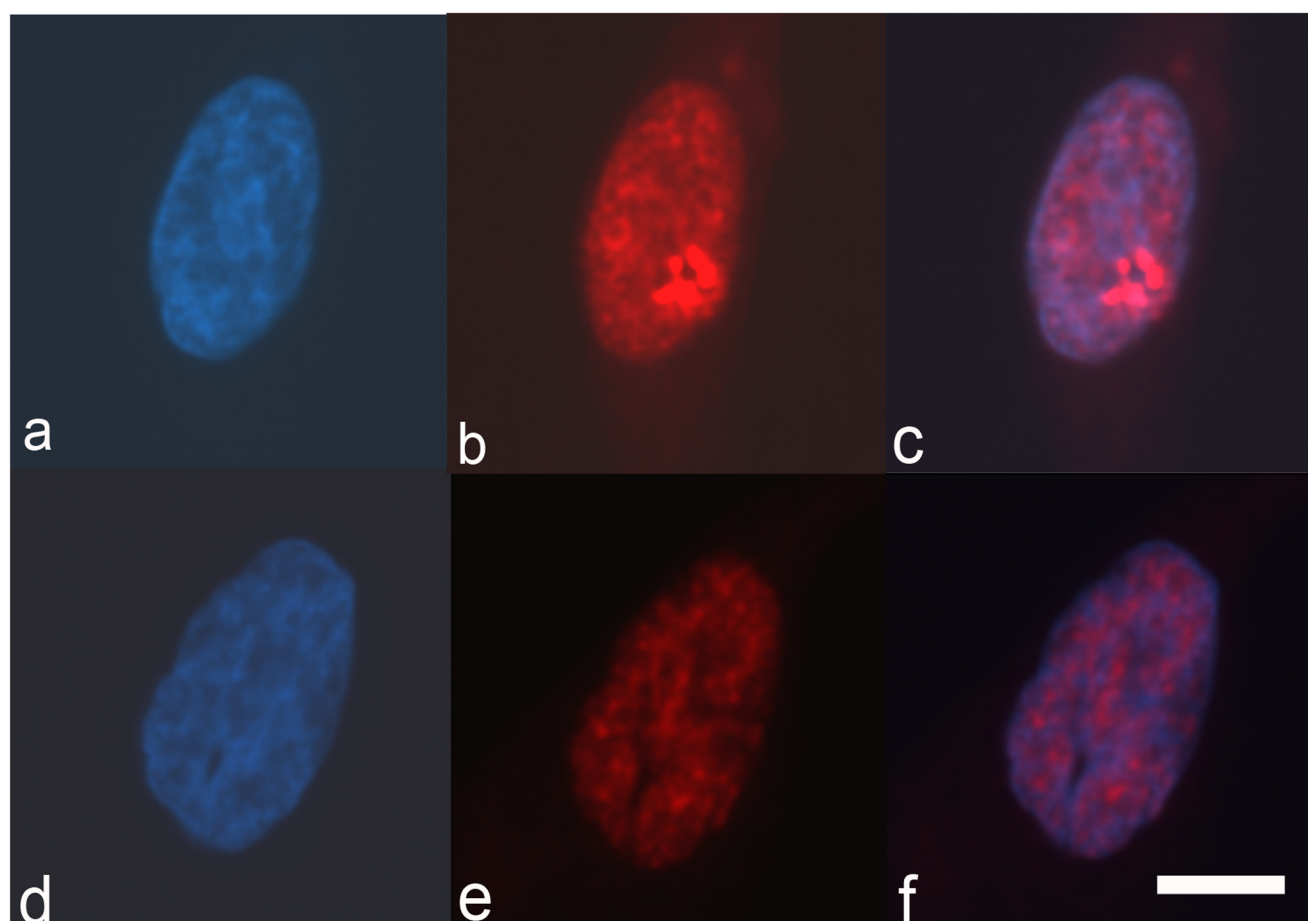

Supplementary Figure 1D

Supplement: Supplementary Figure 1D [file bjc2011117x2.pdf]

A

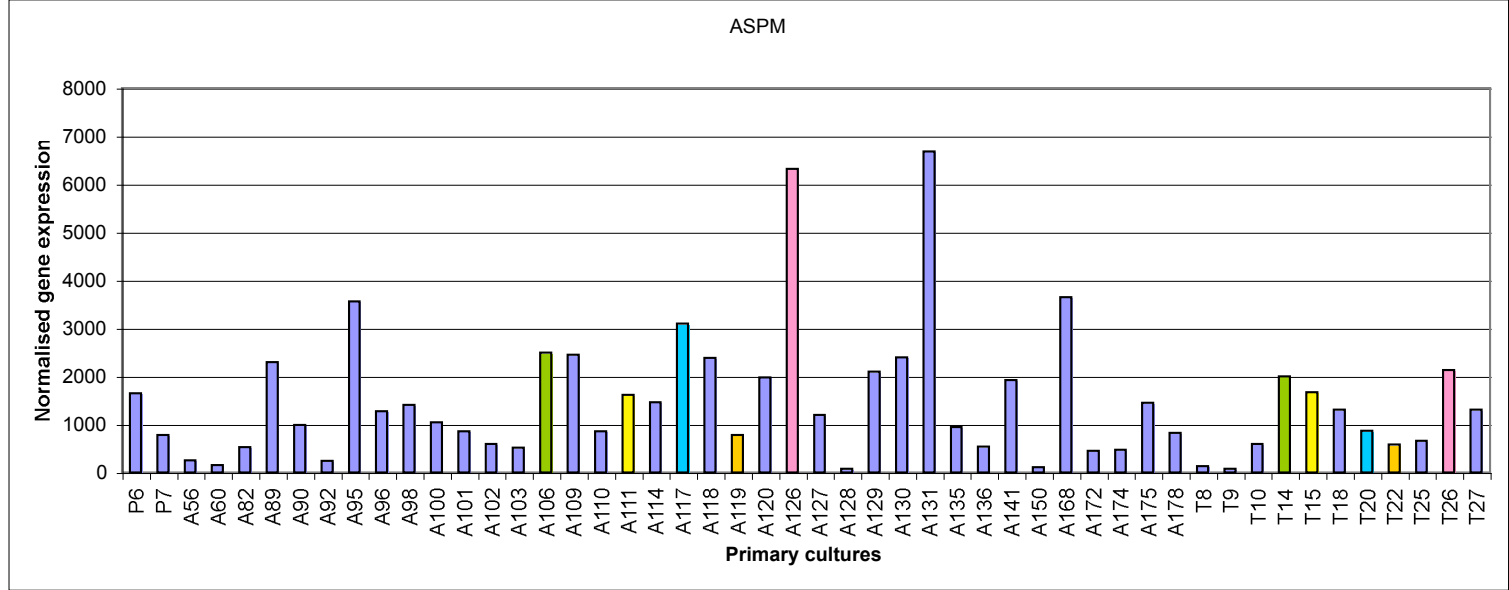

B

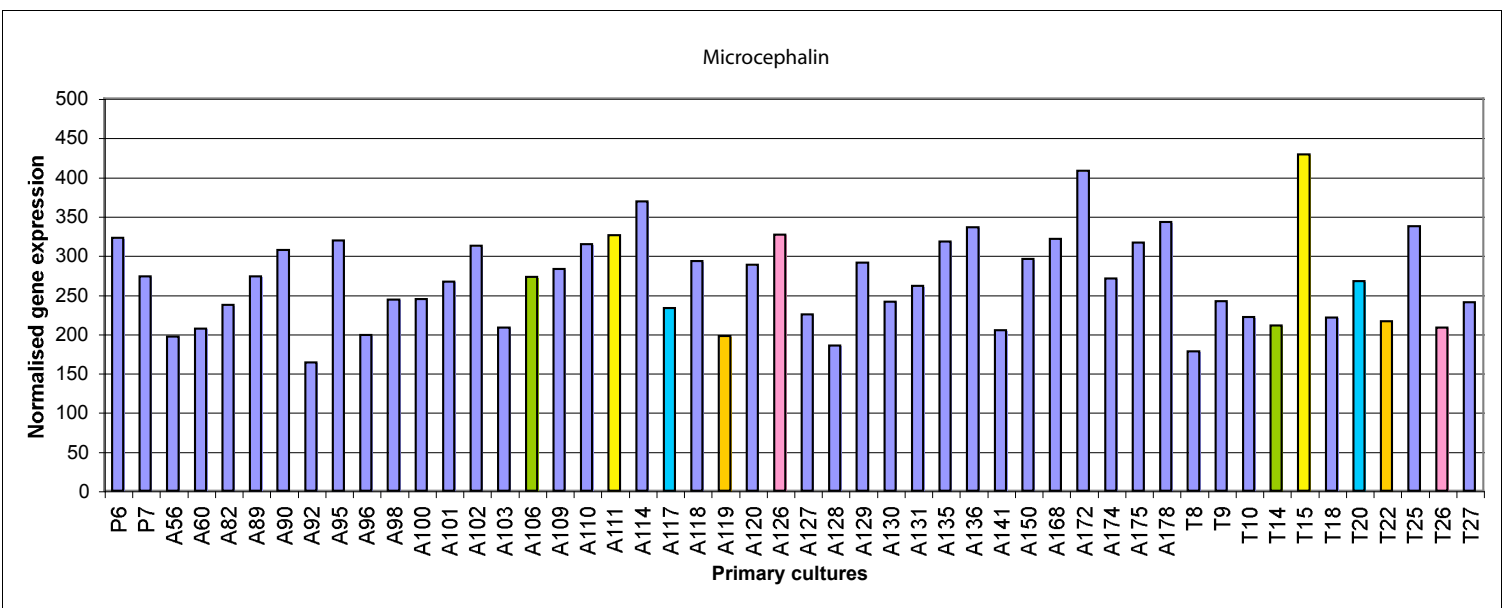

Supplement: Supplementary Figure 2 [file bjc2011117x3.pdf]

A

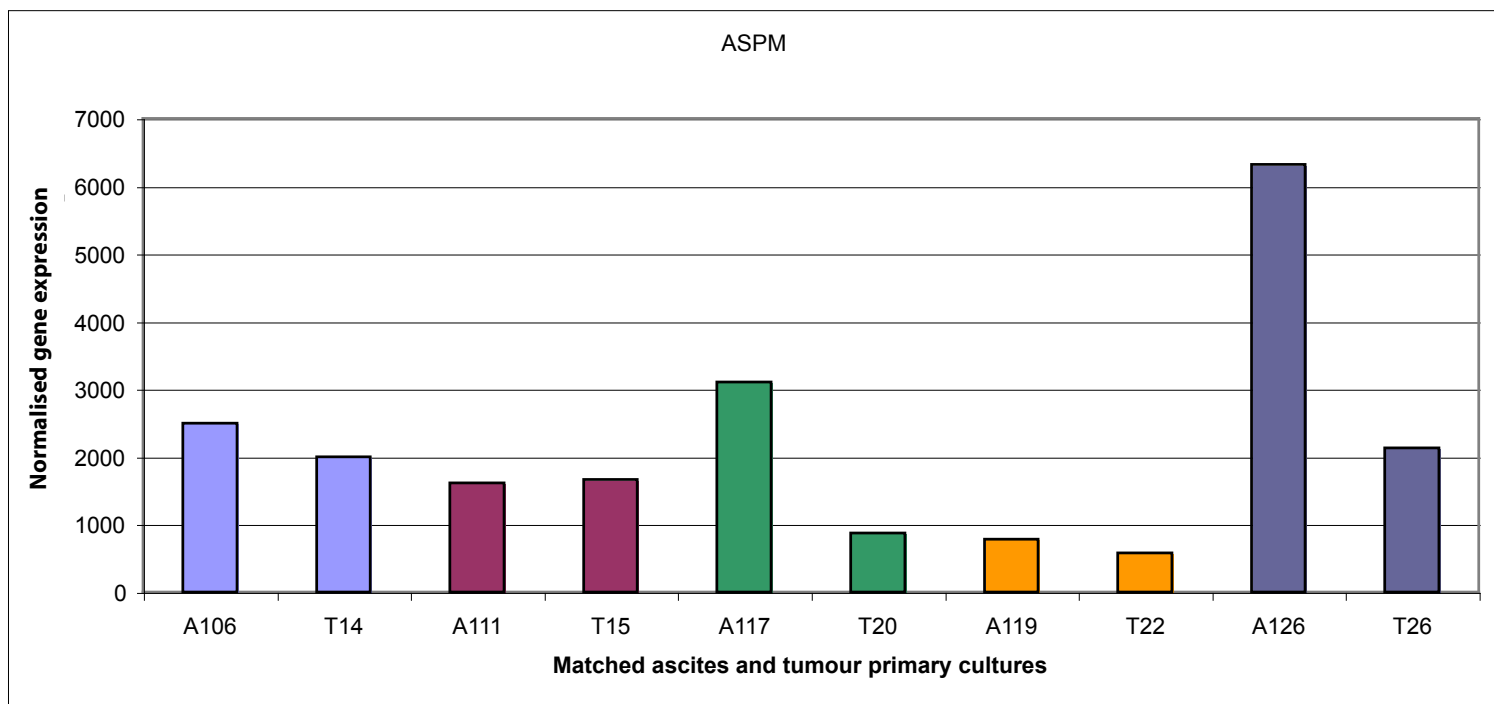

B

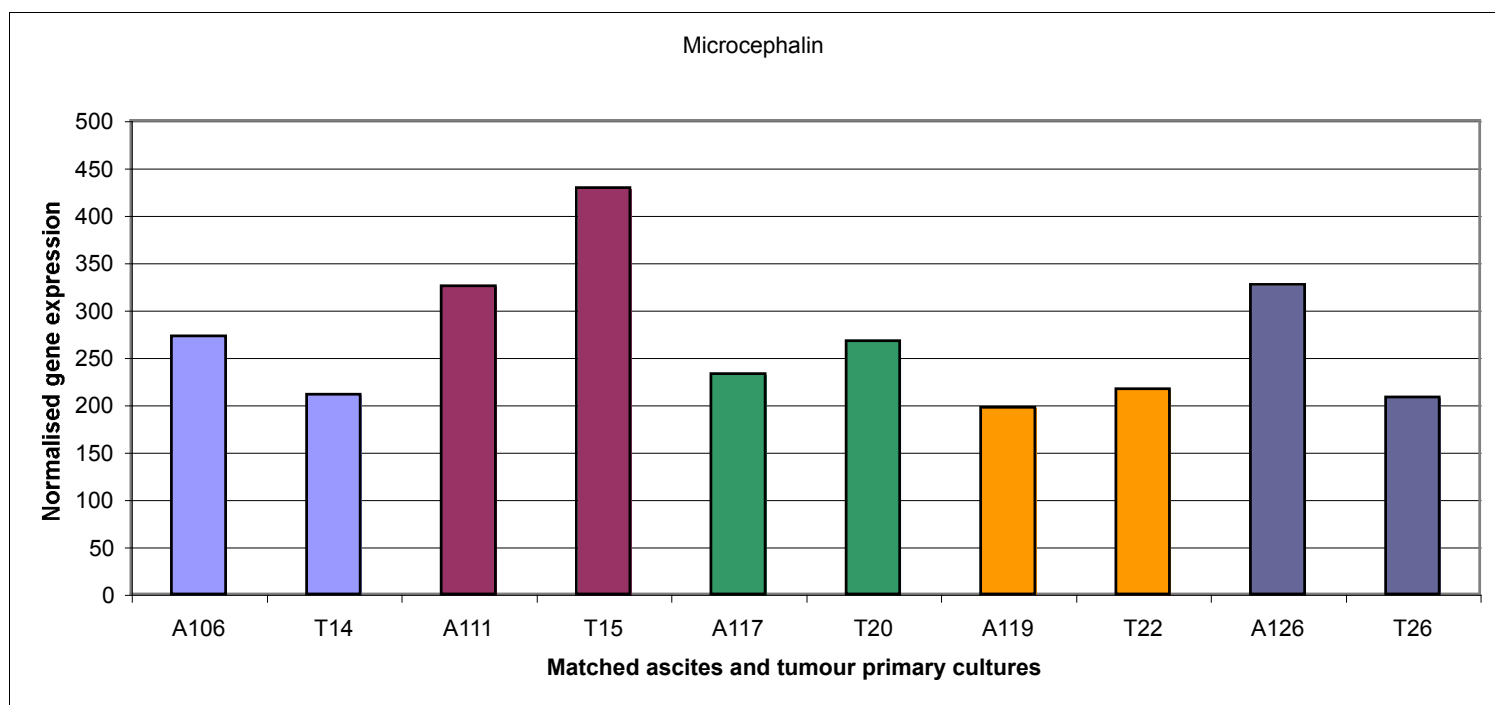

Supplement: Supplementary Figure 3 [file bjc2011117x4.pdf]

A

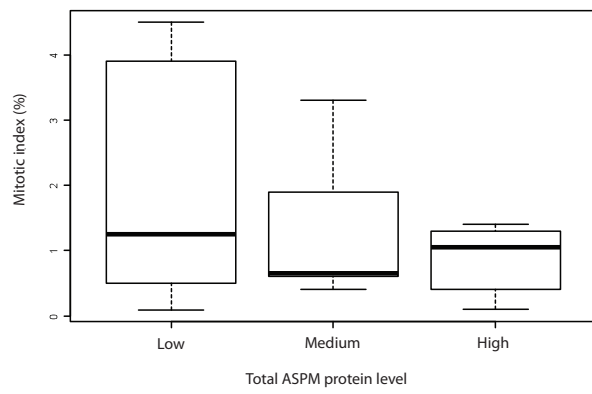

B

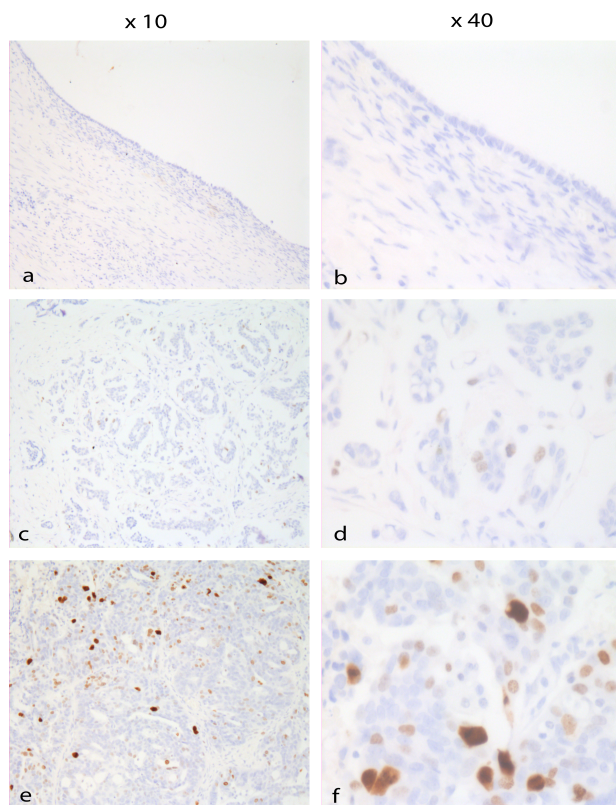

C

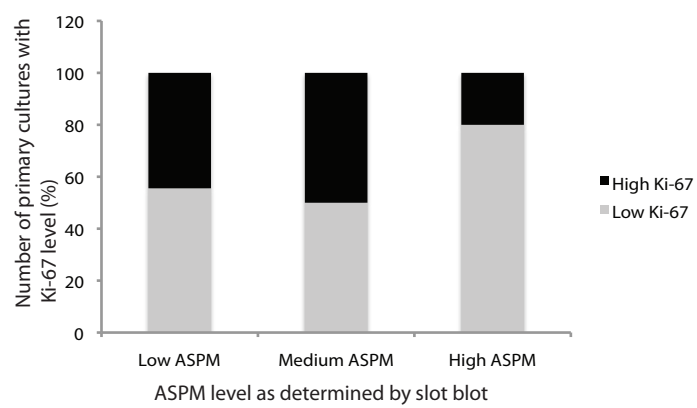

Supplement: Supplementary Figure 4 [file bjc2011117x5.pdf]

A

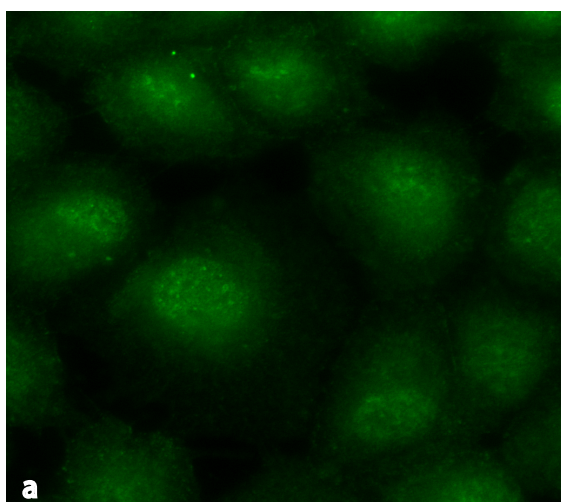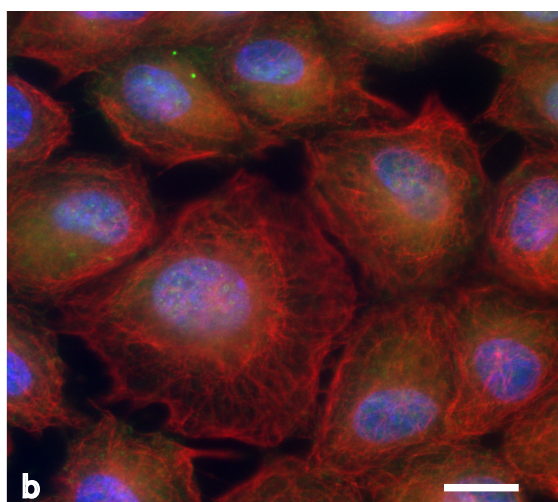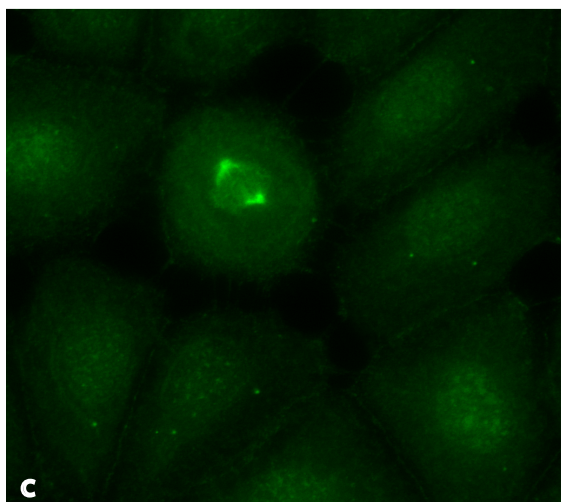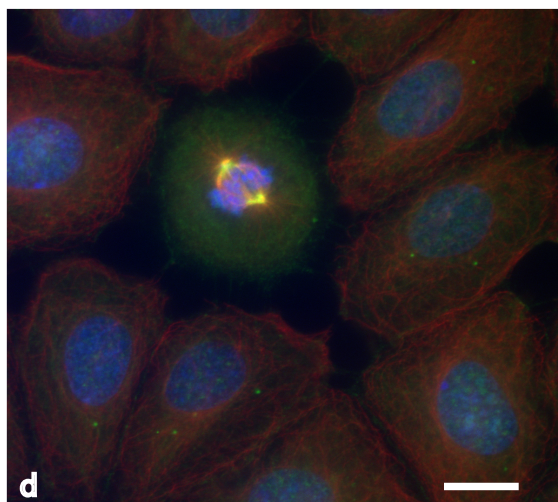

B

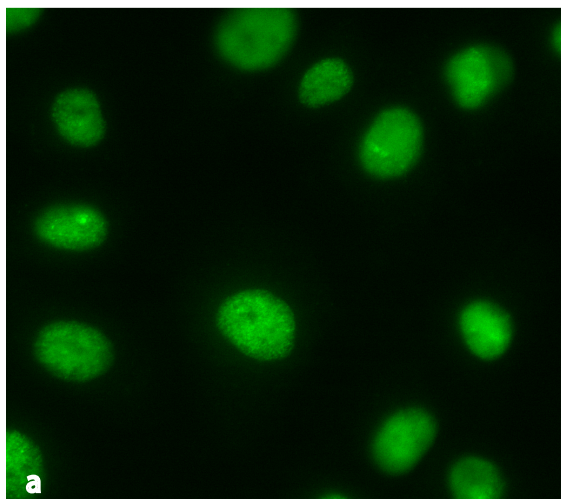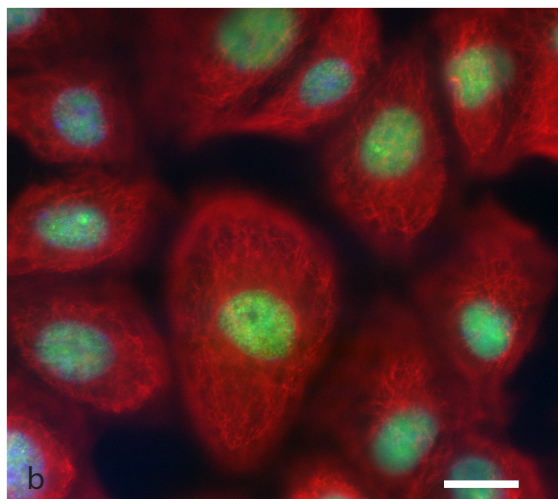

Supplement: Supplementary Figure 5 [file bjc2011117x6.pdf]

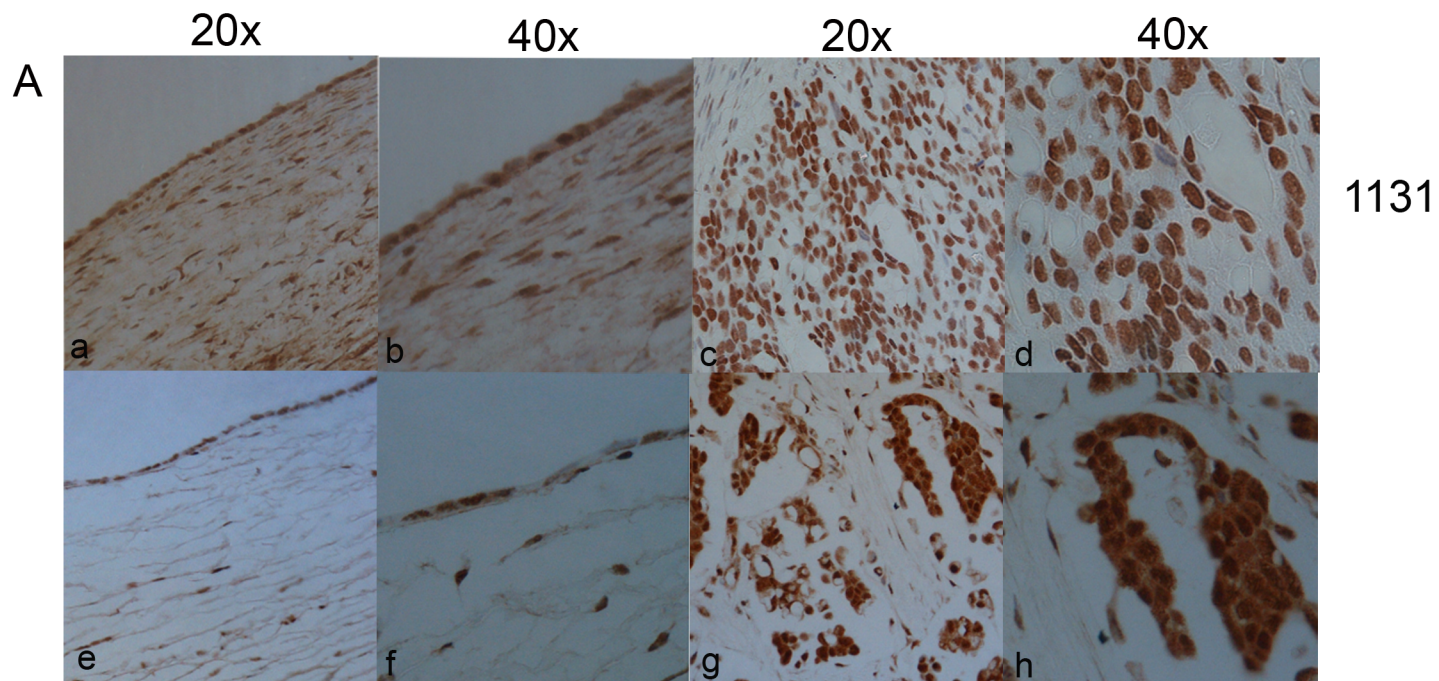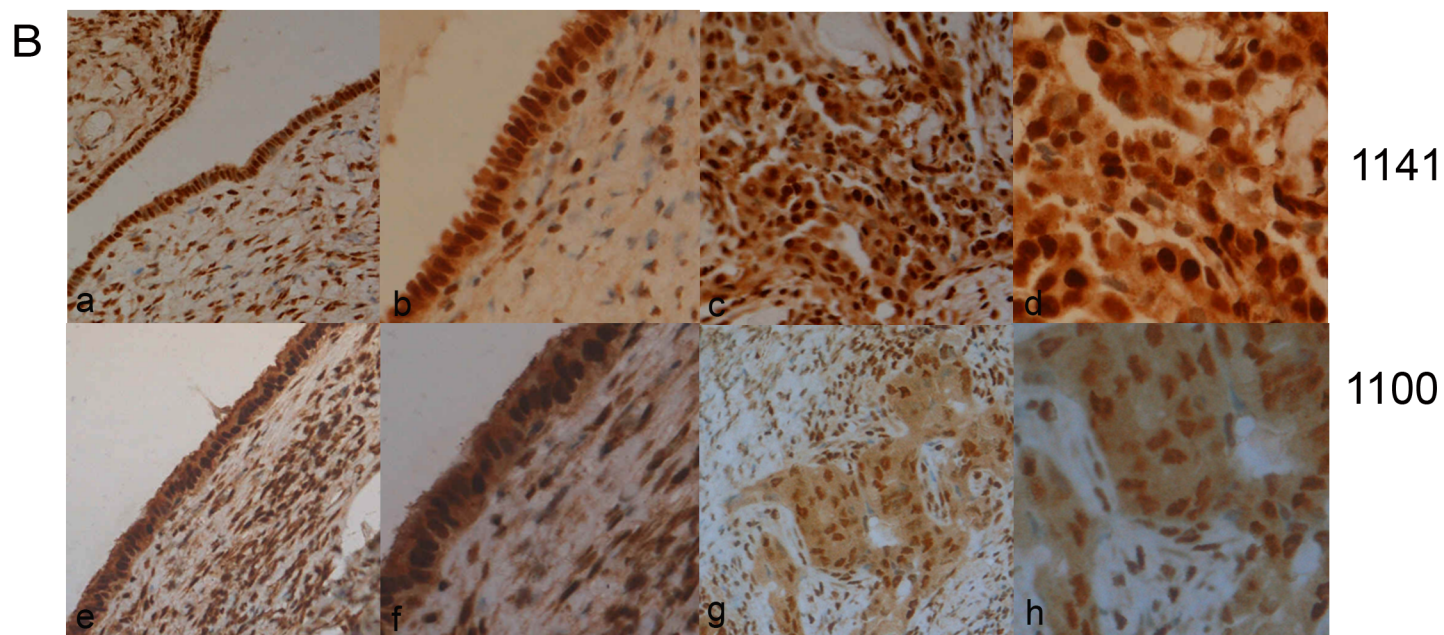

Supplementary Figure 6

Supplement: Supplementary Figure 6 [file bjc2011117x7.pdf]
