# Supplementary material for: ASPM and microcephalin expression in epithelial ovarian cancer correlates with tumour grade and survival
Source: Br J Cancer. 2011 Apr 19;104(10):1602–10. doi: 10.1038/bjc.2011.117 (PMC3101901; doi:10.1038/bjc.2011.117)
Supplement: Supplementary Table 1 [file bjc2011117x8.doc]

**Supplementary Table 1**

**Microcephalin antibodies staining localisation in immunofluorescence**

| **Name & company** | **Raised in** | **Immunogen** | **Localisation** |
| --- | --- | --- | --- |
| Ab2162  Abcam | Rabbit  polyclonal | Synthetic peptide derived from residues 300-400 | Nuclear (plus foci) & cytoplasmic |
| BL1610  Bethyl  Laboratories | Rabbit polyclonal | Protein derived from residues 350-400 | Nuclear, cytoplasmic & centrosomal |
| Microcephalin  IMGENEX | Goat polyclonal | Peptide derived from residues 823-835 HKVCAPENYLLSQ | Nuclear (plus foci) & cytoplasmic |
| APJ-3735  Custom made by Abcam | Chicken Ig | Peptide from residues 110-123 and 624-639  CMQPKDFNFKTPENDK  CDGFKDLIKPHEELKK | No specific staining |
| APJ-258  Custom made by Affinity Research | Rabbit  polyclonal | Peptide derived from residues 411-424  SYDDYFSPDNLKERC | Very weak nuclear (plus foci) & cytoplasmic |
| APJ-254  Custom made by Affinity  Research | Rabbit  polyclonal | Gst-fusion protein derived from residues 9-103 | Very weak nuclear (plus foci) & cytoplasmic |
