# Supplementary material for: ASPM and microcephalin expression in epithelial ovarian cancer correlates with tumour grade and survival
Source: Br J Cancer. 2011 Apr 19;104(10):1602–10. doi: 10.1038/bjc.2011.117 (PMC3101901; doi:10.1038/bjc.2011.117)
Supplement: Supplementary Figures Legends [file bjc2011117x9.doc]

**Supplementary figures**

Supplementary Figure 1. Specificity of the rabbit anti-ASPM antibody 216-1 and the rabbit anti-microcephalin antibody (Abcam ab2612).

To further validate the specificity of the two antibodies used in this study, the affinity purified antibodies were pre-incubated with associated peptides initially used to raise antibodies in rabbits. Antibody was incubated at 4^o^C overnight on a roller with 10x excess of peptide at the final antibody concentration used in immunofluorescence. After incubation, Marvel (0.5%) was added and the solutions were spun at 13,000 rpm for 5 minutes. The supernatants were then applied to coverslips containing methanol fixed cells (ovarian cancer cell lines SKOV-3 and OVCA433). Immunofluorescence was carried out as detailed in the Methods and materials section. Staining was carried out on the same day and the setting for the FITC, TRITC and DAPI channels remained the same throughout imaging to allow direct comparison of staining intensities.

Representative results for antibody staining of SKOV-3 cells after pre-treatment with the peptides and untreated antibody patterns are shown. Similar results were obtained with OVCA433 cells.

A. ASPM nuclear and cytoplasmic staining in SKOV-3 cells (a-d) is greatly reduced after pre-incubation of antibody with peptide (e-f). B. ASPM spindle pole staining (a-d) is greatly reduced when antibody is pre-incubated with the ASPM specific peptide (e-f). Green staining indicates ASPM, red γ-tubulin and blue DAPI. C. Microcephalin nuclear and nuclei focal staining (a-d) is greatly reduced in SKOV-3 cells stained with microcephalin antibody pre-incubated with the microcephalin peptide (e-h). Green staining indicates microcephalin, red γ-tubulin and blue DAPI. All scale bars = 10 μm. D. Transient MCPH1 siRNA transfections were carried out in HeLa cells as described previously (Trimborn *et al*. 2004). Microcephalin nuclear and nuclei focal staining shown in the control siRNA (a-c) is reduced in the *MCPH1* siRNA cells (d-f) stained with microcephalin antibody as described in the Methods and materials. Red staining indicates microcephalin and blue DAPI. All scale bars = 10 μm.

Supplementary Figure 2. Comparison of ASPM (A) and microcephalin (B) mRNA levels in primary cultures derived from pleural effusions (P), malignant ascites (A) and primary tumours (T) from ovarian cancer patients. Data was retrieved from a gene expression microarray analysis, generated with GeneChip HG-U133 Plus 2.0 Arrays (Affymetrix) that was normalised using MAS 5.0. Green, pink bright blue and yellow indicate matching malignant ascites and primary tumour pairs.

Supplementary Figure 3. Comparison of ASPM (A) and microcephalin (B) mRNA levels in matching ascites and primary tumour cultures (grouped in matching pairs) showing high (0.6) and reasonable (0.43) correlations for ASPM and microcephalin mRNA levels respectively. Different cultures indicate matching malignant ascites and primary tumour pairs.

Supplementary Figure 4. A. Correlation of total ASPM levels determined by slot blotting and mitotic activity in the primary cultures. Primary cultures stained with the ASPM antibody 216-1, anti γ-tubulin and DAPI (nuclear stain) were scored at 1000/cells per slide for determination of mitotic activity by recording any mitotic cell visualized by the tubulin and DAPI stain among the interphase cells and expressing the final number as a proportion of all cells scored. This figure (mitotic index) was then correlated statistically to the staining patterns recorded for total ASPM protein levels by slot blotting as described in the main Methods and materials. There was no statistically significant association of the different ASPM levels and mitotic activity. B. Staining patterns of Ki-67 in normal ovarian epithelium (a and b) and tumour sections from patients with associated ascites used to establish primary cultures in this study and for which ASPM slot blot data was available. Eighteen sections containing tumour tissues of patients from which ascitic fluids had been received for establishment of primary cultures were incubated with the Ki-67 antibody (DAKO M7240) (1 hour at 37^o^C) after antigen retrieval in a pressure cooker for 2 minutes. Ki-67 nuclear staining was scored in 5 random fields within a section and expressed as the proportion of tumour cells labeled within the fields containing tumour cells. Examples of staining patterns for the normal epithelium (a,b) and for a low Ki-67 level (c,d) and a high Ki-67 (e,f) are shown. The mean value obtained for all 18 samples (sections) was calculated and a low Ki-67 status was defined as any value falling below the mean and a high Ki-67 as any value above the mean (Kondoh *et al*, 2010). (C). Statistical analysis was carried out to determine a possible association of Ki-67 and ASPM levels. There was no statistically significant association (*p*=0.25).

Supplementary Figure 5. Representative immunofluorescence staining patterns of ASPM and microcephalin in cells derived from a peritoneal wash from a patient with a benign gynaecological condition (1153-A1). A. ASPM nuclear and cytoplasmic staining in interphase cells (a,b) and at the spindle poles in a mitotic cell (c, d).

B. Microcephalin nuclear and nuclei focal staining pattern in interphase cells. Green staining indicates ASPM (a) and microcephalin (b), red γ-tubulin and blue DAPI. All scale bars = 10 μm.

Supplementary Figure 6. ASPM and microcephalin staining patterns in primary cultures are also observed by IHC in associated primary tumours and adjacent normal. Twenty-three sections containing tumour tissues of patients from which ascitic fluids had been received for establishment of primary cultures were incubated with the ASPM antibody (216-1, 1/250) and the microcephalin antibody (Abcam ab2612, 1/300) after antigen retrieval in a pressure cooker for 2 minutes. Nuclear and cytoplasmic staining patterns were scored as no staining (0), weak staining (1), medium staining (2) and strong staining (3). Representative examples for IHC staining patterns are shown.

A. ASPM staining patterns. Both nuclear and cytoplasmic staining was observed. Sample 1131 tumour (c,d) revealed moderate nuclear staining as recorded for primary cultures from the same patient by immunofluorescence. In agreement with this our IHC data suggests that ASPM expression is slightly increased in the tumour (c,d) versus normal (a,b) in these samples. A tumour sample (g,h) where ASPM expression is increased in comparison to associated normal tissue (e,f) is also shown. Although there is no associated ascetic culture for this tumour, this finding supports our observation of increased ASPM expression in cultures generated from tumour ascites versus expression in cultures generated from benign ascites.

B. Microcephalin staining patterns. Nuclear and cytoplasmic staining was observed. Staining observed for sample 1141 normal (a,b) and cancer (c,d) showing strong nuclear staining. Staining for sample 1100 normal (e,f) showing strong nuclear staining and the adjacent cancer (g,h) showing reduced nuclear staining. For comparison to IFAT staining results for samples 1131, 1141, and 1100 please compare with results in bar charts in Figures 3A and B and 5B.

**Supplementary reference**

Kondoh E, Mori S, Yamaguchi K, Baba T, Matsumura N, Cory Barnett J, Whitaker RS, Konishi I, Fujii S, Berchuck A, Murphy SK (2010) Targeting slow-proliferating ovarian cancer cells. *Int J Cancer* **126:** 2448 - 2456

Trimborn M, Bell SM, Felix C, Rashid Y, Jafri H, Griffiths PD, Neumann LM, Krebs A, Reis A, Sperling K, Neitzel H, Jackson AP (2004) Mutations in microcephalin cause aberrant regulation of chromosome condensation. *Am J Hum Genet* **75(2):** 261-266
